# Intake of water and different beverages in adults across 13 countries

**DOI:** 10.1007/s00394-015-0952-8

**Published:** 2015-06-14

**Authors:** I. Guelinckx, C. Ferreira-Pêgo, L. A. Moreno, S. A. Kavouras, J. Gandy, H. Martinez, S. Bardosono, M. Abdollahi, E. Nasseri, A. Jarosz, G. Ma, E. Carmuega, N. Babio, J. Salas-Salvadó

**Affiliations:** Hydration and Health Department, Danone Research, Palaiseau, France; Human Nutrition Unit, Biochemistry Biotechnology Department, Faculty of Medicine and Health Sciences, Hospital Universitari de Sant Joan de Reus, IISPV (Institut d’Investigació Sanitària Pere Virgili), Universitat Rovira i Virgili, C/Sant Llorenç, 21, 43201 Reus, Spain; CIBERobn (Centro de Investigación Biomédica en Red Fisiopatología de la Obesidad y Nutrición), Institute of Health Carlos III, Madrid, Spain; GENUD (Growth, Exercise, NUtrition and Development) Research Group, Faculty of Health Sciences, Universidad de Zaragoza, Saragossa, Spain; Department of Health Human Performance and Recreation, University of Arkansas, Fayetteville, AR USA; British Dietetic Association, Birmingham, UK; School of Life and Medical Services, University of Hertfordshire, Hatfield, UK; RAND Corporation, Santa Monica, CA USA; Hospital Infantil de Mexico Federico Gomez, Mexico City, Mexico; Department of Nutrition, Faculty of Medicine, Universitas Indonesia, Jakarta, Indonesia; Department of Nutrition Research, Faculty of Nutrition Sciences and Food Technology, National Nutrition and Food Technology Research Institute, Shahid Beheshti University of Medical Sciences, Tehran, Iran; National Food and Nutrition Institute, Warsaw, Poland; National Institute for Nutrition and Food Safety, Chinese Center for Disease Control and Prevention, Beijing, China; Department of Nutrition and Food Hygiene, School of Public Health, Peking University, Beijing, China; Centro de Estudios Sobre Nutrición Infantil, Buenos Aires, Argentina

**Keywords:** Water, Beverages, Fluids, Adult population, WHO recommendation, Energy intake, Free sugars

## Abstract

**Purpose:**

To describe the intake of water and all other fluids and to evaluate the proportion of adults exceeding the World Health Organisation (WHO) recommendations on energy intake from free sugar, solely from fluids.

**Methods:**

A total of 16,276 adults (46 % men, mean age 39.8 years) were recruited in 13 countries from 3 continents. A 24-h fluid-specific record over 7 days was used for fluid assessment.

**Results:**

In Spain, France, Turkey, Iran, Indonesia and China, fluid intake was characterised by a high contribution of water (47–78 %) to total fluid intake (TFI), with a mean water intake between 0.76 and 1.78 L/day, and a mean energy intake from fluids from 182 to 428 kcal/day. Between 11 and 49 % of adults exceeded the free sugar WHO recommendations, considering solely fluids. In Germany, UK, Poland and Japan, the largest contributors to TFI were hot beverages (28–50 %) and water (18–32 %). Mean energy intake from fluids ranged from 415 to 817 kcal/day, and 48–62 % of adults exceeded free sugar WHO recommendations. In Mexico, Brazil and Argentina, the contribution of juices and regular sugar beverages (28–41 %) was as important as the water contribution to TFI (17–39 %). Mean energy intake from fluids ranged 565–694 kcal/day, and 60–66 % of the adults exceeded the free sugar WHO recommendation.

**Conclusions:**

The highest volumes recorded in most of the countries were for water, mean energy intake from fluids was up to 694 kcal/day, and 66 % of adults exceeded the free sugar WHO recommendation solely by fluids. Actions to create an environment in favour of water consumption and reduce sugar intake from fluids therefore are warranted.

**Electronic supplementary material:**

The online version of this article (doi:10.1007/s00394-015-0952-8) contains supplementary material, which is available to authorized users.

## Introduction

Total fluid intake (TFI) or its biomarkers have been associated with health outcomes such as the recurrence of kidney stones, renal function, new-onset hyperglycaemia and the prevalence of some components of the metabolic syndrome [1–4]. Therefore, assessing the volume of TFI in populations is important from a public health perspective.

In addition, it is also important to assess the intake of different sources of fluids. During the last decades, the diversity of fluid types with different nutritional composition has increased substantially. These fluids contribute to total intake more than water (e.g. energy, minerals, additives or caffeine), raising the question of their impact on health. In fact, an analysis of NHANES data has demonstrated differences in the risk of chronic kidney disease (CKD) depending on the type of beverages consumed. A low intake of plain water was associated with an increased risk of CKD [adjusted OR for low vs. high intake of plain water = 2.36 (95 % CI 1.10–5.06)], whilst, compared with the highest intake of beverages besides plain water, a low intake was not associated with an increased risk [5].

One explanation for the different health impact of consumption of different fluid types could be due to differences in energy and nutrient content. Recently, different health institutions and nutrition societies have raised concern regarding an excessive intake of energy coming from free sugars, especially present in sugar-sweetened beverages [6]. A meta-analysis of randomised trials and prospective cohort studies showed that among free-living people with ad libitum diets, the intake of free sugars was a determinant of body weight; however, intake was assessed from both food and beverages [7]. In respect of the consumption of sugar-sweetened beverages, there is a substantial scientific evidence relating the frequent intake of this type of beverages and an increased risk of weight gain [8–10], becoming overweight or obese [11–14], developing metabolic syndrome [15–18], type 2 diabetes [19] or other health problems [17, 20, 21] compared with non-regular consumers. This can partly be explained, as described in some cross-sectional and intervention studies, by the observation that frequent consumers of sugar-sweetened beverages had higher total energy intake [22–24]. Given the current obesity pandemic and the estimation by the WHO that diabetes will be the 7th leading cause of death in 2030 [25], it seems relevant to evaluate the daily intake of the different fluid types and their contribution to energy and sugar intake. A recent systematic literature review by Özen et al. [26] reported the fluid intake of adults from 18 different countries. Unfortunately, only 50 % of the 38 surveys included in the review reported the intake of water [26]. In addition, inconsistencies in the study designs, dietary assessment methods used or classification of beverages and age categories limit the comparison of results between countries. Furthermore, most of the surveys designed were originated in the USA and Europe, and it is pertinent in order to have a better understanding of the TFI, types of beverages consumed, and energy and free sugar consumed from beverages to extend the geographical scope of such studies.

Therefore, the aim of the present analysis was: (a) to describe the intake of drinking water and all other type of beverages in adults from 13 countries in three continents including Latin America and Asia, (b) to report energy intake from beverages and (c) to assess the percentage of adults exceeding the WHO recommendations on free sugars intake.

## Methods

### Design and study population

The present analysis gathers original and published data collected in adults (≥18 years) by 13 different cross-sectional surveys. The surveys were conducted in Latin America (Mexico [27], Brazil and Argentina), Europe (Spain [28], France, UK [29], Germany, Poland and Turkey) and Asia (Iran [30], China [31], Indonesia and Japan). Data collection of the individual surveys was performed between 2008 and 2014 by public (Iranian National Nutrition and Food Technology Research Institute, NNFTRI; and Chinese Centre for Disease Control, CDC) and private organisations. The primary objective of these surveys was to assess the intake of drinking water and different types of beverages. A detailed analysis of the volume of TFI (sum of drinking water and beverages of all types) of these 13 surveys can be found elsewhere [32].

A random recruitment of participants was performed in each country either from a database of volunteers for population surveys, or via systematic door-to-door recruitment until the quotas for age, gender, region, habitat and/or socio-economic characteristics in relation to the total country population were met.

Individuals working in company advertising, marketing, market research, the media, the manufacture, distribution and/or sale of water and all kind of beverages were excluded from participation as these individuals might be more aware of their intakes of fluids. Individuals who were not able to read and write in the language of the questionnaire were not eligible to participate in the survey. Having a specific diagnosed disease and/or following a medically prescribed diet were additional exclusion criteria in UK, Iran and China. The surveys in Argentina, Poland and Japan also excluded participants who had taken part in a survey about non-alcoholic drinks in the previous 6 months. Participants who did not complete the full 7 days of the fluid record, participants reporting a mean total daily fluid intake below 0.4 L/day or higher than 6 L/day or those who had participated in a market research study in the previous 6 months were excluded from the analysis. Pregnancy or lactation was not a specific exclusion in the most countries, except in Iran and China.

The effective sample size for the present analysis was 16,276 participants. Individuals who agreed to be part of the survey received detailed information about the survey’s objectives, what was expected from them, and information about the study’s provisions to preserve confidentiality, risks and benefits, and a clear explanation about their option to participate voluntarily or not in the study. After being given a fully informed description of the study, following the principles of informed consent, participants were asked for their oral approval to participate. No monetary incentive was offered for taking part in the study. All data were recorded anonymously. Therefore, participants included in the data set cannot be identified, either directly or through identifiers. The survey protocol of the unpublished surveys was reviewed and approved by the University of Arkansas Review Board (ref. 14-12-376).

### Assessment of the different fluid types

Participants were provided with a 24-h fluid-specific record to collect information on their intake of all fluid types over 7 consecutive days. The 7-day fluid-specific record was presented in the official country language. In all countries except France, Germany and Japan, a paper version of this 7-day fluid-specific record was delivered and explained to the participants during an initial interview at home. After a period of 7 days, the fluid record was collected from the participant’s home by the researcher and checked with the participant. In France, Germany and Japan, participants completed the 7-day fluid-specific record online. On the morning of the first day, these participants received an electronic reminder with written instructions on how to fill in the fluid record. Paper memory cards were made available to the participants so that they could make notes during the day and subsequently complete the fluid record online. Both the paper and online records had the same structure; the participants were asked the type of the beverage, the volume of the intake, whether the beverage was consumed hot or cold, the reason for the intake, and where and when it was consumed. The questionnaire also asked whether the fluid was consumed by itself or with some food, but did not record the food consumed. To assist the participants in estimating how much fluid was consumed, a photographic booklet of standard fluid containers supported the records. The 13 surveys all used this method to assess the fluid intake and were referred to as Liq.In^7^ (abbreviation of Liquid Intake over 7 days).

### Classification of the fluid types

The fluids recorded were classified into: water (tap and bottled water); milk and milk derivatives; hot beverages (coffee, tea and other hot beverages); juices; regular sweetened beverages (RSB) (carbonated and non-carbonated soft drinks, energy drinks, sports drinks and other sugared soft drinks); diet beverages (diet carbonated soft drinks, diet non-carbonated soft drinks, other diet soft drinks); alcoholic drinks and other beverages. A more detailed classification can be found in supplementary Table 1 of this paper. TFI was defined as the sum of all these categories. In UK, Poland, Indonesia and Japan, the intake of diet beverages was very small, and therefore, they were during the first data treatment included in the RSB category. In Argentina, Iran and Indonesia, only non-alcoholic beverages were recorded. In Spain and France, no fluids were classified into the group “Other beverages”.

### Assessment of anthropometric variables

Height in metres (m) and weight in kilograms (kg) were self-reported by participants, except in Poland, Iran and China where these variables were measured. The body mass index (BMI) was calculated (kg/m^2^). In Mexico, Brazil, Argentina, Indonesia and Japan, no anthropometric data were available.

### Calculation of energy and sugar intake from fluids

Energy and sugar intake from different types of beverages was calculated using the updated USDA international food composition tables [33]. Because the quantity consumed of the types of beverages in the category “Other beverages” was very low and these fluids frequently had an unknown food composition, this category was disregarded for the energy and sugar analysis. The percentage of individuals consuming more than 10 % of energy requirements as free sugar, as recommended by WHO, was calculated [6]. WHO strongly recommends the intake of free sugars to less than 10 % of total energy intake and recently even suggested under conditions a further reduction in the intake of free sugars below 5 % of total energy intake [6]. Total energy requirement could not be calculated due to missing data of participants’ weight and height in some countries. Therefore, the food balance sheets from the Food and Agriculture Organisation (FAO) were consulted to retrieve the mean energy intake (kcal/capita/day) of the adult population of the countries included in this analysis, which is accepted for ecological studies [34]. This source, however, contained the mean energy intake for total population, not separated by gender. In order to assess the differences in adherence to the WHO recommendation on free sugar intake between genders, the theoretical recommended daily energy requirement published by the Institute of Medicine was used for total population, but not for each country [35].

### Statistical analysis

Data are presented either as means and 95 % confidence intervals (CI) for continuous variables, or as numbers and percentages for dichotomous variables. The mean intakes are estimated values of all participants, including non-consumers. We compared the distribution of the selected characteristics between groups Student’s *t* tests for continuous variables. All statistical tests were two-tailed, and the significance level was set at *P* < 0.05. A Bonferroni post hoc test was used to correct for multiple comparisons in the online resources 2 and 3. All analyses were performed using the SPSS software version 22.0 (SPSS Inc., Chicago, IL).

## Results

The daily water and beverages intake of 16,276 participants (47 % men) of 13 countries was analysed in the present study. The baseline characteristics of the male and female participants are presented in Table 1. The mean age of the male and female participants was 40.6 (40.3, 40.9) and 39.2 (38.9, 39.5) years, respectively. The mean BMI of the male and female participants was 25.6 (25.4, 25.7) and 25.0 (24.8, 25.1) kg/m^2^, respectively.

**Table 1:** General characteristics of the study population, categorised by country and gender

	*n* (%)	Age (years)	Age categories (years, %)				Weight (kg)	Height (m)	BMI (kg/m^2^)
			18–29	30–39	40–49	≥50			
Mexico, 2012									
Men	574 (38)	38.6 (37.4, 40.0)	35.5	18.3	17.2	28.9	ND	ND	ND
Woman	924 (62)	38.3 (37.5, 39.2)	33.0	22.3	21.0	23.7	ND	ND	ND
Brazil, 2008									
Men	941 (49)	34.5 (33.8, 35.2)	39.5	25.3	25.4	9.8	ND	ND	ND
Woman	983 (51)	34.7 (34.0, 35.4)	36.8	27.7	25.5	10.0	ND	ND	ND
Argentina, 2009									
Men	241 (47)	37.1 (35.3, 38.8)	38.6	21.6	18.3	21.6	ND	ND	ND
Woman	266 (56)	37.8 (36.2, 39.4)	36.5	20.7	17.3	25.6	ND	ND	ND
Spain, 2012									
Men	630 (51)	42.9 (41.8, 44.0)	18.6	25.4	23.3	32.7	78.8 (77.8, 79.8)	1.7 (1.7, 1.7)	26.1 (25.8, 26.4)
Woman	610 (49)	43.0 (41.9, 44.1)	19.5	21.5	26.2	32.8	65.2 (64.3, 66.1)	1.6 (1.6, 1.6)	25.1 (24.7, 25.4)
France, 2012									
Men	804 (52)	47.6 (46.5, 48.6)	15.7	16.8	18.4	49.1	80.5 (79.6, 81.5)	1.7 (1.7, 1.8)	26.1 (25.8, 26.4)
Woman	730 (48)	41.5 (40.5, 42.5)	22.2	23.2	24.5	30.1	65.5 (64.4, 66.6)	1.6 (1.6, 1.6)	24.2 (23.8, 24.6)
UK, 2010									
Men	371 (41)	46.3 (44.7, 47.9)	16.7	20.8	20.2	42.3	ND	ND	28.8 (28.2, 29.5)
Woman	526 (59)	42.2 (41.0, 43.4)	19.6	26.4	25.3	28.7	ND	ND	25.9 (25.3, 26.5)
Germany, 2012									
Men	856 (45)	44.1 (43.2, 44.9)	16.4	20.1	26.5	37.0	81.6 (80.3, 82.8)	1.8 (1.8, 1.8)	25.8 (25.4, 26.2)
Woman	1012 (54)	41.9 (41.2, 42.7)	17.5	22.3	30.6	29.5	77.0 (75.9, 78.2)	1.7 (1.7, 1.7)	27.3 (26.8, 27.7)
Poland, 2014									
Men	517 (49)	46.0 (44.5, 47.4)	19.5	19.0	19.1	42.4	82.8 (81.6, 84.0)	1.8 (1.7, 1.8)	26.6 (26.3, 27.0)
Woman	545 (51)	46.2 (44.8, 47.6)	19.4	20.7	16.5	43.3	70.1 (68.9, 71.3)	1.6 (1.6, 1.7)	25.5 (25.1, 25.9)
Turkey, 2011									
Men	488 (51)	34.4 (33.4, 35.3)	37.7	27.3	24.2	10.9	75.9 (74.8, 77.0)	1.7 (1.7, 1.7)	25.0 (24.7, 25.4)
Woman	473 (49)	34.3 (33.4, 35.3)	38.5	27.9	22.6	11.0	65.8 (64.6, 67.1)	1.6 (1.6, 1.6)	25.0 (24.5, 25.5)
Iran, 2011									
Men	283 (49)	37.3 (35.8, 38.8)	36.7	24.0	19.4	19.8	79.3 (77.7, 81.0)	1.7 (1.7, 1.8)	25.8 (25.3, 26.2)
Woman	289 (51)	36.5 (35.1, 37.9)	35.3	28.0	20.1	16.6	63.9 (62.6, 65.3)	1.6 (1.6, 1.6)	24.9 (24.3, 25.4)
China, 2010									
Men	733 (50)	39.5 (38.6, 40.4)	24.7	25.2	25.8	24.3	67.4 (66.6, 68.2)	1.7 (1.7, 1.7)	23.2 (23.0, 23.5)
Woman	733 (50)	39.3 (38.5, 40.2)	24.7	25.6	25.8	23.9	55.9 (55.3, 56.5)	1.6 (1.6, 1.6)	22.1 (21.9, 22.3)
Indonesia, 2012									
Men	444 (32)	35.5 (34.3, 36.7)	39.4	25.7	16.9	18.0	ND	ND	ND
Woman	922 (68)	35.1 (34.4, 35.8)	39.3	28.5	17.6	14.6	ND	ND	ND
Japan, 2009									
Men	698 (51)	ND	26.6	27.4	21.5	24.5	ND	ND	ND
Woman	683 (49)	ND	26.1	27.1	21.1	25.8	ND	ND	ND
Total population^a^									
Men	7580 (47)	40.6 (40.3, 40.9)	27.0	22.8	22.0	28.3	77.9 (77.5, 78.4)	1.7 (1.7, 1.7)	25.6 (25.4, 25.7)
Woman	8696 (53)	39.2 (38.9, 39.5)	28.0	24.8	23.3	23.9	67.0 (66.6, 67.5)	1.6 (1.6, 1.6)	25.0 (24.8, 25.1)

Data expressed as mean (95 % CI) or percentage

*BMI* body mass index, *ND* no data

^a^Include only those countries with data available on the presented characteristics

The daily intakes of the different beverage types are presented in Table 2. Among the different fluid types, the highest volumes were observed for water intake, which ranged from 0.27 L/day in Japan to 1.78 L/day in Indonesia. The second type of fluid consumed in terms of volume was hot beverages, with a daily intake ranging from 0.12 L/day in Mexico to 1.03 L/day in UK. RSB was the third mostly fluid consumed with a daily intake ranging from 0.10 L/day in China to 0.57 L/day in Mexico.

**Table 2:** Total daily consumption of different types of beverages (L/day) for total population

	Water	Milk and derivates	Hot beverages	Juices	Regular sweetened beverages	Diet beverages	Alcoholic beverages	Other beverages	Total fluid intake
Mexico (*n* = 1498)	0.70 (0.66, 0.73)	0.19 (0.18, 0.20)	0.12 (0.11, 0.13)	0.18 (0.16, 0.19)	0.57 (0.54, 0.59)	0.02 (0.01, 0.02)	0.03 (0.02, 0.04)	0.00 (0.00, 0.00)	1.81 (1.76, 1.86)
Brazil (*n* = 1924)	0.83 (0.80, 0.86)	0.21 (0.20, 0.22)	0.31 (0.28, 0.33)	0.48 (0.46, 0.50)	0.23 (0.21, 0.24)	0.01 (0.00, 0.01)	0.15 (0.13, 0.17)	0.00 (0.00, 0.01)	2.22 (2.17, 2.27)
Argentina (*n* = 507)	0.39 (0.35, 0.43)	0.16 (0.15, 0.18)	0.92 (0.86, 0.98)	0.27 (0.24, 0.31)	0.37 (0.31, 0.42)	0.19 (0.16, 0.22)	ND	0.00 (0.00, 0.00)	2.30 (2.22, 2.39)
Spain (*n* = 1240)	1.01 (0.97, 1.05)	0.10 (0.09, 0.11)	0.30 (0.29, 0.32)	0.09 (0.08, 0.10)	0.16 (0.14, 0.17)	0.04 (0.03, 0.05)	0.20 (0.18, 0.22)	ND	1.90 (1.86, 1.95)
France (*n* = 1534)	0.76 (0.73, 0.78)	0.06 (0.06, 0.07)	0.39 (0.37, 0.41)	0.06 (0.06, 0.06)	0.12 (0.11, 0.13)	0.03 (0.02, 0.03)	0.13 (0.12, 0.14)	ND	1.56 (1.52, 1.59)
UK (*n* = 897)	0.51 (0.46, 0.55)	0.09 (0.08, 1.00)	1.03 (0.98, 1.07)	0.12 (0.1, 0.13)	0.37 (0.33, 0.40)	ND^a^	0.20 (0.18, 0.22)	0.00 (0.00, 0.01)	2.32 (2.26, 2.37)
Germany (*n* = 1868)	0.79 (0.75, 0.82)	0.29 (0.27, 0.31)	0.69 (0.66, 0.72)	0.18 (0.17, 0.20)	0.26 (0.24, 0.28)	0.01 (0.01, 0.01)	0.25 (0.23, 0.26)	0.01 (0.01, 0.01)	2.47 (2.43, 2.52)
Poland (*n* = 1062)	0.46 (0.44, 0.48)	0.08 (0.07, 0.09)	0.73 (0.71, 0.75)	0.09 (0.08, 0.10)	0.17 (0.15, 0.18)	ND^a^	0.10 (0.09, 0.11)	0.01 (0.00, 0.01)	1.64 (1.60, 1.67)
Turkey (*n* = 961)	1.04 (1.00, 1.09)	0.06 (0.05, 0.07)	0.51 (0.48, 0.54)	0.12 (0.11, 0.14)	0.20 (0.18, 0.22)	0.00 (0.00, 0.00)	0.01 (0.01, 0.02)	0.25 (0.23, 0.27)	2.21 (2.15, 2.27)
Iran (*n* = 572)	0.96 (0.91, 1.02)	0.17 (0.16, 0.19)	0.51 (0.48, 0.53)	0.07 (0.06, 0.07)	0.13 (0.11, 0.14)	0.01 (0.01, 0.02)	ND	0.07 (0.06, 0.08)	1.92 (1.86, 1.99)
China (*n* = 1466)	0.96 (0.92, 0.99)	0.10 (0.10, 0.11)	0.45 (0.42, 0.49)	0.02 (0.02, 0.02)	0.10 (0.09, 0.11)	ND^a^	0.09 (0.08, 0.10)	0.03 (0.03, 0.03)	1.76 (1.71, 1.81)
Indonesia (*n* = 1366)	1.78 (1.73, 1.84)	0.05 (0.04, 0.05)	0.26 (0.24, 0.27)	0.02 (0.01, 0.02)	0.17 (0.15, 0.20)	ND^a^	ND	0.01 (0.01, 0.02)	2.28 (2.23, 2.34)
Japan (*n* = 1381)	0.27 (0.25, 0.29)	0.08 (0.07, 0.09)	0.75 (0.73, 0.78)	0.06 (0.05, 0.06)	0.11 (0.10, 0.11)	ND^a^	0.21 (0.19, 0.23)	0.01 (0.01, 0.02)	1.50 (1.46, 1.53)
Total population (*n* = 16,276)	0.82 (0.81, 0.84)	0.13 (0.13, 0.14)	0.49 (0.49, 0.50)	0.14 (0.14, 0.15)	0.22 (0.21, 0.22)	0.02 (0.02, 0.02)	0.12 (0.12, 0.12)	0.02 (0.02, 0.02)	1.98 (1.96, 1.99)

Data expressed as mean (95 % CI)

*ND* no data

^a^In case of UK, Poland, China, Indonesia and Japan, the intake of diet beverages was included in the regular sweetened beverages category

**Fig. 1:**
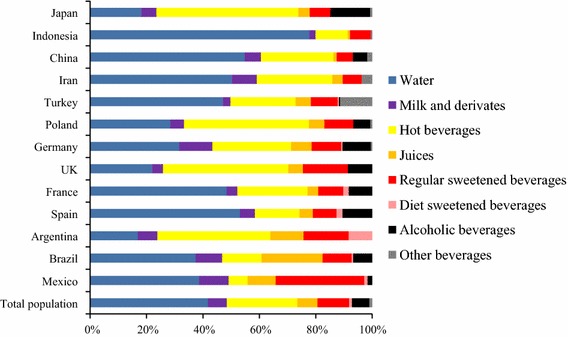
Contribution of the different types of beverages to TFI stratified by country

Significant gender differences were inconsistently observed across countries for the daily intake of different types of beverages (supplementary Table 2). Water intake was significantly higher among women than men in Germany, Turkey and the total sample, whereas water intake was lower among women than men in Brazil. Women had a significantly higher milk intake then men in Brazil, Germany and the total sample. A higher intake among women than men was also observed for hot beverages in Mexico, Spain, France and Poland. The significant difference in RSB intake between genders was also inconsistent across countries. In Brazil, Spain and Germany, women consumed less RSB than men, whereas in France and China women consumed more RSB than men. The significant gender effect on diet beverages was consistent, yet only present in two countries: women consumed more diet beverages then men in Spain and France. The mean intake of alcoholic beverages was significantly higher among men than women in Mexico, Brazil, Spain, France, Germany, Poland and the total sample. Figure 1 represents the contribution (%) of the different fluid types to TFI. Countries with similar contribution patterns can be identified. Indonesia, China, Spain, Iran, Turkey and France are countries with the largest contribution of water to TFI, ranging from 47 to 78 %. The second largest contributor to TFI, in all these countries, was hot beverages. A different pattern was observed in Mexico and Brazil. In these countries, the contribution of RSB and juices to TFI was as large as the water contribution to TFI. This was also the case of Argentina, where the contribution of juices and RSB is larger than the water contribution; however, hot beverages are the primary contributor to TFI in this country. A high contribution of hot beverages to TFI was also observed among Germany, Poland and UK. However, unlike in Argentina, the contribution of water to TFI was larger than the contribution of RSB and juices. The contribution of water, juices, RSB and alcoholic beverages to TFI was comparable in these three countries (Germany, Poland and UK). The largest contribution of hot beverages (50 %) and alcoholic beverages (14 %) to TFI was observed in Japan.

Table 3 shows the mean energy intake from fluids. Total mean energy intake of total fluid ranged from a minimum of 182 kcal/day in Indonesia to a maximum of 817 kcal/day in Germany. In the total sample, the highest mean energy intake came from the consumption of milk and derivatives, followed by alcoholic beverages and then hot beverages. In Germany, Brazil, Iran, China and Spain, the milk and derivatives consumption represented the highest energy intake of all fluid types (299, 220, 182, 110 and 108 kcal/day, respectively). In France and Japan, the highest energy intake came from alcoholic beverages (95 and 159 kcal/day, respectively), whereas in UK, Poland and Turkey, hot beverages delivered the highest energy intake (205, 146 and 102 kcal/day, respectively). In Mexico and Indonesia, the highest energy intake from fluids came from RSB (232 and 74 kcal/day, respectively). Significant gender difference in energy intake provided by the different fluid types was also observed (Supplementary Table 3). In the total sample, men had a significantly higher mean energy intake from RSB and alcoholic beverages than women. Women on the other hand had a higher energy intake provided by milk and derivatives than men.

**Table 3:** Energy intake (in kcal) of different types of fluid intake by country and total population

	Milk and derivates	Hot beverages	Juices	Regular sweetened beverages	Diet beverages	Alcoholic beverages	Total fluid intake
Mexico (*n* = 1498)	200 (189, 211)	25 (23, 27)	80 (74, 86)	232 (220, 243)	3 (2, 3)	25 (19, 30)	565 (547, 583)
Brazil (*n* = 1924)	220 (208, 232)	62 (57, 66)	216 (207, 225)	82 (76, 89)	1 (1, 1)	113 (98, 129)	694 (672, 716)
Argentina (*n* = 507)	168 (153, 183)	184 (172, 197)	123 (108, 139)	147 (124, 170)	27 (23, 31)	ND	649 (622, 676)
Spain (*n* = 1240)	108 (96, 119)	61 (58, 64)	42 (38, 47)	63 (56, 69)	5 (4, 7)	149 (135, 164)	428 (408, 447)
France (*n* = 1534)	66 (59, 73)	78 (75, 82)	27 (25, 29)	52 (48, 57)	4 (3, 5)	95 (87, 103)	329 (318, 340)
UK (*n* = 897)	98 (86, 110)	205 (196, 215)	54 (48, 60)	151 (138, 164)	ND^a^	148 (132, 164)	656 (633, 679)
Germany (*n* = 1868)	299 (279, 318)	138 (133, 143)	81 (75, 88)	108 (100, 116)	2 (1, 2)	187 (174, 199)	817 (792, 842)
Poland (*n* = 1062)	83 (76, 91)	146 (142, 150)	41 (38, 45)	70 (65, 76)	ND^a^	76 (68, 84)	415 (401, 428)
Turkey (*n* = 961)	65 (56, 74)	102 (97, 107)	55 (48, 62)	85 (77, 94)	0 (0, 1)	8 (5, 12)	314 (298, 330)
Iran (*n* = 572)	182 (167, 196)	102 (97, 107)	29 (26, 32)	50 (44, 56)	2 (1, 2)	ND	365 (347, 383)
China (*n* = 1466)	110 (103, 117)	91 (83, 98)	9 (8, 11)	41 (36, 45)	ND^a^	69 (59, 79)	320 (305, 336)
Indonesia (*n* = 1366)	48 (41, 54)	52 (48, 55)	8 (6, 9)	74 (64, 84)	ND^a^	ND	182 (170, 194)
Japan (*n* = 1381)	84 (77, 90)	151 (146, 155)	26 (24, 29)	40 (36, 44)	ND^a^	159 (146, 172)	460 (444, 475)
Total population (*n* = 16,276)	140 (137, 144)	99 (97, 101)	64 (62, 66)	91 (88, 93)	2 (2, 3)	91 (87, 94)	490 (483, 496)

Data expressed as mean (95 % CI)

*ND* no data

^a^In case of UK, Poland, China, Indonesia and Japan, the intake of diet beverages was included in the regular sweetened beverages category

**Fig. 2:**
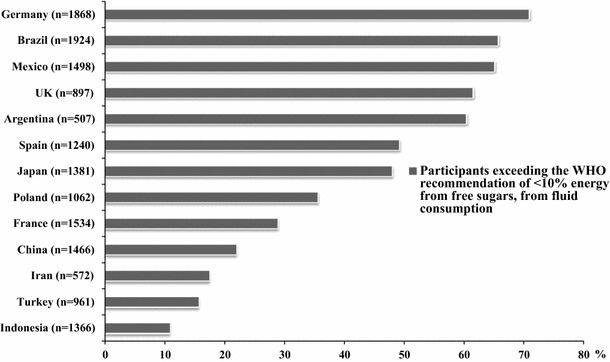
Participants exceeding WHO recommendations for free sugar (<10 % of energy), considering only fluid intake

Figure 2 shows the proportion of participants exceeding the WHO recommendation on energy intake provided by free sugar, solely by the intake of fluids. The highest proportion of adults exceeding the WHO recommendation was observed in Germany (70.9 %), followed by Brazil (65.7 %), Mexico (65.1 %), UK (61.5 %) and Argentina (60.4 %), whereas the lowest proportion was observed in Indonesia (10.9 %). Considering all countries together, 44.5 % of the population exceeded the WHO recommendations on energy intake provided by free sugar, solely by fluids.

## Discussion

The aim of the present analysis was to collate and describe the intake of water and all other fluids of adults of 13 cross-sectional surveys, which used the same 7-day fluid-specific record. This unique compilation of national surveys conducted in large sample of participants from different countries demonstrated that not only the volume, but also the contribution of the different fluid types to TFI varied substantially between countries. Nevertheless, some countries that seemed to be geographically linked share similar patterns. The fluid intake of the countries relatively close to the Mediterranean Sea (Spain, France, Turkey and Iran) and also the two Asian countries (Indonesia and China) was characterised by a high contribution of water to TFI, ranging from 47 to 78 %. In North European countries (UK, Poland and Germany), the highest contribution to TFI came from hot beverages. The fluid intake of the three countries of Latin America was characterised by a high contribution of juices and RSB, which is as important as the contribution of water to TFI. Due to these substantial inter-geographical area differences in fluid intake contribution, the pooled data of all countries should be interpreted with caution. Identifying factors that could explain these observed between-country differences was not the aim of the current analysis, yet several hypotheses can be made. One of the possible factors explaining the between-country differences is climate and seasonality. Studies analysing seasonality of fluid intake indicated that temperature and seasons affected both volume and choice of beverages type [36]. However, Tani et al. [37] reported that in China an increase of 1 °C in the mean outdoor air temperature on the survey day was associated with an increased intake of water from fluids by only 8.4 mL/day (*P* < 0.0001) and that the influence of humidity was non-significant. Therefore, it seems unlikely that the differences observed between countries in volumes of beverage can be explained by climate alone. The impact of seasonality on the reported fluid intake between countries cannot be evaluated in this study, because in each survey data collection took place once during a period of the year with a mild climate (spring or autumn). Other factors to take into consideration when assessing the between-country variability are cultural and traditional habits, which unfortunately were not evaluated in the present study.

The data in the current analysis are in line with data reported in other published surveys. The fluid intake pattern with a high contribution of water to TFI characteristically for the Mediterranean countries was also reported in France by Bellisle et al. [39] and in Italy by Leclercq et al. [38]. Even though Bellisle et al. [39] reported a lower TFI and a higher intake of milk and alcoholic beverages in French adults than observed in our French survey, a pattern characterised by a relatively high contribution of water (43 %) and hot beverages (20 %) was also observed. Furthermore, a high contribution of water to TFI (58 % in men and 67 % in women) was also observed among Italian adults [38]. Although Italy was not included in the current analysis, this beverage pattern was similar than that we observed among the countries relatively close to the Mediterranean Sea.

The intake of the North European countries in the current surveys was characterised by a high intake of hot beverages. The National Nutritional Survey II assessed food intake of 15,371 German adults confirmed a similar contribution of hot beverages (21 %) to TFI, even though they reported higher volumes of all fluid types and a higher contribution of water to TFI (42 vs. 32 % in the present study) than in the current analysis. The contribution of juices and RSB to TFI observed in the present analysis was also different compared with others surveys [40]. These differences can be explained in part because in the present study these fluid types were split; however, the combined contribution (juices plus RSB) of 20 % is comparable to the 17 % observed in previous studies. For the UK, two previously published surveys reported TFI volumes and energy intake provided by beverages among adults that were in line with our observations [22, 41]. A survey performed in another North European country not included in the current analysis, Finland, also showed a fluid intake pattern characterised by a high contribution of water (34 and 51 % for men and women, respectively) and hot beverages (39 and 37 % for men and women, respectively) to TFI [42].

In Latin American countries, publications reporting fluid intake in adults were mainly focussed on the intake of caloric beverages and covered only the Mexican population [43, 44]. These two Mexican surveys described volumes of intake for the different fluid categories comparable to those in the current analysis. However, in both studies mean energy intake from fluids (372 and 382 kcal/day/per capita, respectively) was estimated to be lower than that estimated in the present study, which could be probably explained by a different classification of the beverages and the use of different food composition data.

The results obtained in the present international survey highlight the need to educate adults about the nutritional composition of the different fluids. As observed in previous studies, an accurate education programme and public health actions would be effective to encourage regular consumers to decrease their RSB consumption with water or other non-caloric beverages, in order to decrease the risk of chronic disease such as type 2 diabetes [23, 45] or overweight/obesity [7]. Attention should also be paid to fruit juices, because adult individuals still perceive beverages such as squashes, fruit lemonades and fruit sodas as a healthy option, and they should be advised about the low fruit content and the higher amounts of sugar [46]. In this present analysis, juices and RSB were separated into two different categories, because the nutritional composition is different. 100 % fruit juices could potentially contribute to daily vitamin and antioxidants intake [47]. However, regarding sugar content, RSB and juices are comparable; therefore, an increased intake should not be encouraged.

Several limitations of the fluid surveys or the current analysis need to be discussed. As often happens in nutritional research, the self-reported surveys collecting the intake of fluids are open to potential bias due to the over- or under-reporting of certain fluid types. This limitation can also be related to the current analysis, even though the same 7-day fluid-specific record was used in all the surveys. Another limitation was that the classification of diet beverages was not performed in the same way in all countries and alcoholic beverages were not recorded in some surveys, which limited the comparison between countries. Sugar and energy content per 100 mL of fluid type was used for the estimation of the energy and sugar consumed from each beverage in all countries. These were an approximate estimation of the reality, since the same fluid type of the same brand can have a different nutritional composition depending on the country. Additionally, sugar or other ingredients added by the consumer to the fluids were not taken into account for the calculation of energy intake. Therefore, the energy intake from fluids is likely to be underestimated. For the evaluation of the percentage of energy provided by free sugar, total energy intake had to be estimated since no food data were collected. The lack of food data also limited the interpretation of the data on energy intake from fluids. However, evidence suggested that a fluid-specific record might more accurately estimate fluid intake compared with a food and fluid record [48]. Since the primary aim of all 13 surveys included was to assess fluid intake, the preference was given to record fluids only. Due to the lack of anthropometric data of the participants in certain countries, the calculations had to be based on population means of energy intake. Nevertheless, this assumption was considered to be acceptable in epidemiological studies since the individual surveys aimed at collecting data from a nationally large sample of individuals and also because the energy intake data for the food balance sheets were recorded during the same year of the fluid surveys.

Despite these methodological limitations, this analysis has several strengths. This analysis is unique as it collated data of 13 surveys with relatively large sample sizes and an equal distribution between both genders. The compilation also contains original data from countries, which previously had no internally published fluid intake data available. The third strength is the use of the same 7-day fluid-specific diary in all the surveys that was also supported by a photographic booklet to limit the self-reporting error. Finally, the intake of drinking water and all other fluids were reported, in the 13 surveys except alcoholic beverages in three countries. This is rather exceptional as shown in the systematic review by Özen et al. [26].

In conclusion, the current study shows that intake volumes of the different fluid types differ considerably between countries, but these differences in the contribution to TFI are modest between countries of the same geographical area. Even though the highest volume consumed was recorded for drinking water, the mean energy intake from fluids was higher than expected due to the high consumption of RSB and fruit juices (reached up to 694 kcal/day of energy intake on average). Since the proportion of adults exceeding the WHO recommendation for energy intake provided by free sugars ranged from 11 up to 70.9 %, educating adults about the nutritional composition of the different fluids seems a pertinent step but not only one in terms of public health. Health authorities and food industry should take complementary actions to promote fluids with low sugar content and to create an environment favouring water consumption.

## Electronic supplementary material

Supplementary material 1 (DOCX 31 kb)

Supplementary material 2 (DOCX 41 kb)

Supplementary material 3 (DOCX 38 kb)
