# Supplementary material for: Intake of water and different beverages in adults across 13 countries
Source: Eur J Nutr. 2015 Jun 14;54(Suppl 2):45–55. doi: 10.1007/s00394-015-0952-8 (PMC4473281; doi:10.1007/s00394-015-0952-8)
Supplement: Supplementary file 1 — Supplementary material 1 (DOCX 31 kb) [file 394_2015_952_MOESM1_ESM.docx]

**Supplementary file 1.** Classification of the fluid types

| **Classification  of fluids** | **Detailed Fluid types** |
| --- | --- |
| Water | Still water, unflavored sparkling water, tap/filtered/boiled water |
| Milk and derivatives | Low fat and full fat milk, fermented milk, ready-to-drink milk, flavored milk, yogurt milk, *atole/champurado*, raw milk, powder milk, powder/syrup flavored milk, fruit shake with milk, cocoa compound with milk |
| Hot beverages | Coffee, Coffee from coffee maker (e.g.: homemade coffee, dolce gusto, others), powder coffee, instant coffee, vending machine coffee, restaurant/franchise coffee, homemade hot/cold tea (from tea bags or leaves), infusions (herbal) |
| Juices | Packages fruits & vegetable juices (packaged juices (fruits or vegetables), packaged orangeade, packaged nectars, Eskimos/smoothies), *aguas frescas*, natural fruits & vegetables juices (natural fruit/vegetable juices, restaurant lemonade/orangeade), sugarcane juice |
| Regular sweetened beverages | Carbonated sweet beverages (CSB): cola carbonated drinks, flavored carbonated drinks flavored sparkling water, tonic, soda, flavored packaged water, flavored waters made with powder or concentrate/syrup, fruit shake with powder, ready to drink tea, ready to drink ice tea, sports drinks (e.g. Aquarius), vitamin/ functional drinks (fiber, vitamin and cooling drinks, e.g. C1000 Vitamin Lemon), energy drinks, cocoa prepared with water |
| Diet beverages | Diet sweet beverages (packaged light juices, diet/light/zero cola carbonated drinks, diet/light/zero flavored carbonated drinks) |
| Alcoholic beverages | Beer, lager, beer mix drinks, wine, champagne, aperitifs and digestives, packaged/canned alcoholic beverages, spirits, cocktails |
| Other beverages | - Beverages identified by participant as “other than listed above” - Packaged soy drinks, traditional Indonesian drinks, Jamu (Indonesia), Agua de arroz (Mexico), diet drinks as meal replacement(slim fast), Ready to drink soy based juice, Ayran (Turkey) |
