# Supplementary material for: Intake of water and different beverages in adults across 13 countries
Source: Eur J Nutr. 2015 Jun 14;54(Suppl 2):45–55. doi: 10.1007/s00394-015-0952-8 (PMC4473281; doi:10.1007/s00394-015-0952-8)
Supplement: Supplementary file 2 — Supplementary material 2 (DOCX 41 kb) [file 394_2015_952_MOESM2_ESM.docx]

**Supplementary file 2.** Total daily consumption of different types of fluids (L/day) stratified by gender

|  | Water | Milk and derivates | Hot beverages | Juices | Regular sweetened beverages | Diet beverages | Alcoholic beverages | Other beverages | Total Fluid Intake |
| --- | --- | --- | --- | --- | --- | --- | --- | --- | --- |
| Mexico |  |  |  |  |  |  |  |  |  |
| Men (n=574) | 0.64 (0.59, 0.69)^a^ | 0.20 (0.18, 0.22) | 0.11 (0.10, 0.12) ^a^ | 0.18 (0.15, 0.20) | 0.58 (0.54, 0.63) | 0.01 (0.01, 0.02) | 0.05 (0.03, 0.06) ^a^ | 0.00 (0.00, 0.00) | 1.77 (1.70, 1.85) |
| Women (n=924) | 0.73 (0.69, 0.78) | 0.18 (0.17, 0.20) | 0.14 (0.12, 0.15) | 0.18 (0.16, 0.19) | 0.56 (0.52, 0.59) | 0.02 (0.01, 0.03) | 0.02 (0.01, 0.03) | 0.00 (0.00, 0.00) | 1.84 (1.77, 1.90) |
| Brazil |  |  |  |  |  |  |  |  |  |
| Men (n=941) | 0.87 (0.83, 0.92) ^a^ | 0.20 (0.18, 0.21) ^a^ | 0.28 (0.25, 0.31) | 0.49 (0.46, 0.52) | 0.27 (0.24, 0.29) ^a^ | 0.00 (0.00, 0.01) | 0.22 (0.18, 0.25) ^a^ | 0.00 (0.00, 0.00) ^a^ | 2.34 (2.27, 2.42) ^a^ |
| Women (n=983) | 0.78 (0.74, 0.82) | 0.22 (0.21, 0.24) | 0.33 (0.29, 0.37) | 0.47 (0.44, 0.49) | 0.19 (0.18, 0.21) | 0.01 (0.00, 0.01) | 0.08 (0.06, 0.11) | 0.01 (0.00, 0.01) | 2.10 (2.03, 2.17) |
| Argentina |  |  |  |  |  |  |  |  |  |
| Men (n=241) | 0.36 (0.30, 0.42) | 0.16 (0.14, 0.18) | 0.96 (0.87, 1.05) | 0.27 (0.21, 0.32) | 0.39 (0.31, 0.47) | 0.17 (0.13, 0.20) | ND | 0.00 (0.00, 0.01) | 2.32 (2.20, 2.44) |
| Women (n=266) | 0.41 (0.35, 0.46) | 0.16 (0.14, 0.18) | 0.89 (0.80, 0.98) | 0.28 (0.24, 0.32) | 0.34 (0.27, 0.42) | 0.21 (0.17, 0.26) | ND | 0.00 (0.00, 0.00) | 2.29 (2.17, 2.42) |
| Spain |  |  |  |  |  |  |  |  |  |
| Men (n=630) | 0.97 (0.92, 1.02) | 0.11 (0.09, 0.13) | 0.26 (0.24, 0.28) ^a^ | 0.10 (0.08, 0.12) | 0.18 (0.15, 0.20) ^a^ | 0.03 (0.01, 0.04) ^a^ | 0.29 (0.25, 0.32) ^a^ | ND | 1.94 (1.87, 2.00) |
| Women (n=610) | 1.05 (1.00, 1.10) | 0.09 (0.08, 0.11) | 0.35 (0.32, 0.37) | 0.09 (0.08, 0.10) | 0.13 (0.11, 0.15) | 0.05 (0.03, 0.06) | 0.11 (0.09, 0.12) | ND | 1.87 (1.80, 1.93) |
| France |  |  |  |  |  |  |  |  |  |
| Men (n=804) | 0.75 (0.72, 0.79) | 0.07 (0.06, 0.08) | 0.35 (0.33, 0.37) ^a^ | 0.06 (0.06, 0.07) | 0.11 (0.10, 0.12) ^a^ | 0.02 (0.02, 0.03) ^a^ | 0.17 (0.16, 0.19) ^a^ | ND | 1.55 (1.50, 1.60) |
| Women (n=730) | 0.76 (0.73, 0.80) | 0.06 (0.05, 0.07) | 0.44 (0.41, 0.47) | 0.06 (0.05, 0.06) | 0.14 (0.12, 0.15) | 0.04 (0.03, 0.04) | 0.07 (0.06, 0.08) | ND | 1.57 (1.52, 1.62) |
| UK |  |  |  |  |  |  |  |  |  |
| Men (n=371) | 0.49 (0.43, 0.56) | 0.09 (0.07, 0.10) | 1.00 (0.92, 1.07) | 0.11 (0.09, 0.13) | 0.37 (0.33, 0.42) | ND | 0.17 (0.15, 0.20) | 0.00 (0.00, 0.00) | 2.24 (2.15, 2.32) ^a^ |
| Women (n=526) | 0.52 (0.46, 0.57) | 0.10 (0.08, 0.11) | 1.05 (0.99, 1.11) | 0.12 (0.11, 0.14) | 0.36 (0.32, 0.40) | ND | 0.21 (0.18, 0.24) | 0.00 (0.00, 0.01) | 2.37 (2.30, 2.45) |
| Germany |  |  |  |  |  |  |  |  |  |
| Men (n=856) | 0.75 (0.70, 0.81) ^a^ | 0.25 (0.22, 0.28) ^a^ | 0.70 (0.66, 0.74) | 0.18 (0.15, 0.20) | 0.28 (0.25, 0.32) ^a^ | 0.01 (0.01, 0.01) | 0.32 (0.29, 0.38) ^a^ | 0.01 (0.01, 0.02) | 2.51 (2.45, 2.57) |
| Women (n=1012) | 0.81 (0.77, 0.86) | 0.32 (0.29, 0.34) | 0.68 (0.64, 0.72) | 0.18 (0.16, 0.20) | 0.23 (0.21, 0.26) | 0.01 (0.01, 0.02) | 0.19 (0.17, 0.21) | 0.01 (0.01, 0.01) | 2.45 (2.39, 2.50) |
| Poland |  |  |  |  |  |  |  |  |  |
| Men (n=517) | 0.48 (0.45, 0.52) | 0.08 (0.07, 0.09) | 0.71 (0.68, 0.73) ^a^ | 0.10 (0.08, 0.11) | 0.18 (0.16, 0.20) | ND | 0.15 (0.13, 0.17) ^a^ | 0.00 (0.00, 0.01) | 1.70 (1.66, 1.75) ^a^ |
| Women (n=545) | 0.44 (0.40, 0.47) | 0.08 (0.07, 0.09) | 0.75 (0.73, 0.78) | 0.09 (0.08, 0.10) | 0.16 (0.14, 0.17) | ND | 0.05 (0.04, 0.06) | 0.00 (0.00, 0.01) | 1.57 (1.53, 1.62) |
| Turkey |  |  |  |  |  |  |  |  |  |
| Men (n=488) | 0.98 (0.92, 1.04) ^a^ | 0.05 (0.04, 0.07) | 0.50 (0.47, 0.54) | 0.13 (0.11, 0.15) | 0.20 (0.18, 0.23) | 0.00 (0.00, 0.01) | 0.01 (0.00, 0.02) | 0.26 (0.23, 0.28) | 2.15 (2.07, 2.24) |
| Women (n=473) | 1.11 (1.05, 1.17) | 0.07 (0.06, 0.08) | 0.52 (0.48, 0.55) | 0.11 (0.09, 0.13) | 0.20 (0.17, 0.23) | 0.00 (0.00, 0.00) | 0.01 (0.00, 0.02) | 0.25 (0.22, 0.28) | 2.27 (2.17, 2.37) |
| Iran |  |  |  |  |  |  |  |  |  |
| Men (n=283) | 0.96 (0.89, 1.03) | 0.18 (0.16, 0.20) | 0.53 (0.49, 0.56) | 0.07 (0.06, 0.09) | 0.12 (0.11, 0.14) | 0.01 (0.01, 0.02) | ND | 0.06 (0.05, 0.07) | 1.92 (1.83, 2.02) |
| Women (n=289) | 0.97 (0.89, 1.04) | 0.17 (0.15, 0.19) | 0.49 (0.45, 0.53) | 0.06 (0.05, 0.07) | 0.13 (0.11, 0.16) | 0.02 (0.01, 0.02) | ND | 0.08 (0.06, 0.09) | 1.92 (1.83, 2.02) |
| China |  |  |  |  |  |  |  |  |  |
| Men (n=733) | 0.98 (0.92, 1.03) | 0.11 (0.10, 0.12) | 0.45 (0.40, 0.51) | 0.02 (0.02, 0.02) | 0.09 (0.07, 0.10) ^a^ | ND | 0.10 (0.08, 0.12) | 0.03 (0.02, 0.03) | 1.78 (1.71, 1.85) |
| Women (n=733) | 0.94 (0.89, 0.99) | 0.10 (0.09, 0.11) | 0.45 (0.40, 0.50) | 0.02 (0.02, 0.02) | 0.11 (0.09, 0.12) | ND | 0.09 (0.07, 0.10) | 0.03 (0.03, 0.04) | 1.75 (1.68, 1.81) |
| Indonesia |  |  |  |  |  |  |  |  |  |
| Men (n=444) | 1.82 (1.72, 1.92) | 0.04 (0.03, 0.05) | 0.25 (0.22, 0.28) | 0.02 (0.01, 0.02) | 0.20 (0.15, 0.24) | ND | ND | 0.02 (0.01, 0.02) | 2.33 (2.23, 2.43) |
| Women (n=922) | 1.76 (1.70, 1.83) | 0.05 (0.04, 0.05) | 0.26 (0.24, 0.28) | 0.02 (0.01, 0.02) | 0.17 (0.14, 0.20) | ND | ND | 0.01 (0.01, 0.02) | 2.26 (2.19, 2.32) |
| Japan |  |  |  |  |  |  |  |  |  |
| Men (n=698) | 0.27 (0.24, 0.29) | 0.08 (0.07, 0.08) | 0.74 (0.71, 0.78) | 0.06 (0.05, 0.06) | 0.09 (0.08, 0.10) | ND | 0.22 (0.19, 0.24) | 0.01 (0.01, 0.02) | 1.47 (1.42, 1.52) |
| Women (n=683) | 0.28 (0.25, 0.31) | 0.08 (0.07, 0.09) | 0.76 (0.73, 0.80) | 0.07 (0.06, 0.08) | 0.11 (0.09, 0.12) | ND | 0.20 (0.18, 0.23) | 0.02 (0.01, 0.02) | 1.52 (1.47, 1.57) |
| Total population |  |  |  |  |  |  |  |  |  |
| Men (n=7580) | 0.80 (0.78, 0.81) ^a^ | 0.13 (0.12, 0.13) ^a^ | 0.49 (0.48, 0.50) | 0.14 (0.14, 0.15) | 0.21 (0.20, 0.22) ^a^ | 0.02 (0.01, 0.02) | 0.16 (0.15, 0.17) ^a^ | 0.02 (0.02, 0.03) | 1.97 (1.95, 1.99) |
| Women (n=8696) | 0.85 (0.83, 0.86) | 0.14 (0.13, 0.14) | 0.50 (0.49, 0.51) | 0.14 (0.14, 0.15) | 0.22 (0.21, 0.23) | 0.02 (0.02, 0.02) | 0.09 (0.08, 0.09) | 0.02 (0.02, 0.02) | 1.98 (1.96, 2.00) |

Data expressed as mean (95% CI). Abbreviation: ND, no data. ^a^ P-value <0.05 for men vs. women, with a Bonferroni correction applied during the post-hoc test
