# Supplementary material for: Intake of water and different beverages in adults across 13 countries
Source: Eur J Nutr. 2015 Jun 14;54(Suppl 2):45–55. doi: 10.1007/s00394-015-0952-8 (PMC4473281; doi:10.1007/s00394-015-0952-8)
Supplement: Supplementary file 3 — Supplementary material 3 (DOCX 38 kb) [file 394_2015_952_MOESM3_ESM.docx]

**Supplementary file 3.** Energy intake from different types of fluid intake (kcal/day) stratified by gender

|  | Milk and derivates | Hot beverages | Juices | Regular sweetened beverages | Diet beverages | Alcoholic beverages | Total Fluid Intake |
| --- | --- | --- | --- | --- | --- | --- | --- |
| Mexico |  |  |  |  |  |  |  |
| Men (n=574) | 212 (193, 231) | 22 (19, 24) ^a^ | 80 (70, 91) | 239 (220, 258) | 2 (1, 3) | 35 (25, 46) ^a^ | 590 (560, 620) ^a^ |
| Women (n=924) | 192 (179, 206) | 27 (24, 30) | 80 (72, 87) | 229 (214, 244) | 3 (2, 4) | 18 (12, 24) | 549 (526, 572) |
| Brazil |  |  |  |  |  |  |  |
| Men (n=941) | 207 (189, 224) ^a^ | 57 (51, 62) | 222 (209, 236) | 96 (86, 106) ^a^ | 1 (0, 1) | 165 (139, 190) ^a^ | 748 (714, 783) ^a^ |
| Women (983) | 232 (216, 249) | 66 (59, 74) | 210 (198, 222) | 69 (62, 76) | 1 (1, 1) | 64 (46, 82) | 642 (614, 670) |
| Argentina |  |  |  |  |  |  |  |
| Men (n=241) | 169 (147, 192) | 191 (173, 209) | 121 (97, 146) | 158 (124, 192) | 23 (18, 29) | ND | 662 (620, 705) |
| Women (n=266) | 168 (147, 188) | 178 (160, 195) | 125 (107, 144) | 136 (106, 167) | 30 (24, 36) | ND | 637 (603, 671) |
| Spain |  |  |  |  |  |  |  |
| Men (n=630) | 116 (97, 135) | 52 (48, 56) ^a^ | 45 (38, 53) | 72 (61, 82) ^a^ | 4 (2, 6) ^a^ | 216 (192, 241) ^a^ | 505 (473, 537) ^a^ |
| Women (n=610) | 100 (87, 112) | 70 (65, 74) | 39 (34, 44) | 52 (44, 59) | 7 (5, 9) | 80 (67, 94) | 348 (328, 368) |
| France |  |  |  |  |  |  |  |
| Men (n=804) | 71 (60, 81) | 70 (66, 74) ^a^ | 28 (25, 31) | 47 (41, 53) ^a^ | 3 (2, 4) ^a^ | 131 (119, 143) ^a^ | 358 (341, 375) ^a^ |
| Women (n=730) | 61 (51, 71) | 88 (82, 93) | 26 (23, 29) | 58 (51, 65) | 5 (4, 6) | 55 (48, 63) | 297 (283, 311) |
| UK |  |  |  |  |  |  |  |
| Men (n=371) | 90 (75, 105) | 200 (184, 215) | 50 (41, 59) | 154 (134, 173) | ND | 130 (110, 151) | 624 (591, 657) ^a^ |
| Women (n=526) | 104 (87, 120) | 210 (197, 222) | 56 (48, 65) | 148 (130, 166) | ND | 160 (138, 183) | 678 (646, 711) |
| Germany |  |  |  |  |  |  |  |
| Men (n=856) | 262 (233, 291) ^a^ | 140 (132, 148) | 80 (70, 90) | 120 (107, 133) ^a^ | 1 (1, 2) | 239 (218, 261) ^a^ | 841 (804, 878) |
| Women (n=1012) | 330 (303, 357) | 136 (129, 143) | 83 (74, 92) | 98 (87, 109) | 2 (2, 3) | 142 (129, 156) | 797 (764, 830) |
| Poland |  |  |  |  |  |  |  |
| Men (n=517) | 80 (70, 91) | 141 (136, 147) ^a^ | 44 (38, 50) | 76 (67, 84) | ND | 115 (101, 129) ^a^ | 453 (431, 475) ^a^ |
| Women (n=545) | 86 (75, 97) | 150 (145, 155) | 39 (35, 44) | 66 (59, 72) | ND | 39 (33, 45) | 378 (362, 394) |
| Turkey |  |  |  |  |  |  |  |
| Men (n=488) | 57 (45, 70) | 101 (94, 108) | 60 (50, 70) | 86 (75, 97) | 1 (0, 1) | 9 (3, 15) | 300 (278, 322) |
| Women (n=473) | 72 (59, 86) | 103 (96, 111) | 50 (41, 59) | 85 (72, 97) | 0 (0, 1) | 8 (3, 13) | 329 (306, 352) |
| Iran |  |  |  |  |  |  |  |
| Men (n=283) | 186 (165, 206) | 106 (98, 113) | 29 (25, 34) | 45 (37, 52) | 2 (1, 2) | ND | 368 (343, 394) |
| Women (n=289) | 178 (157, 199) | 98 (91, 105) | 29 (24, 33) | 55 (46, 65) | 2 (1, 3) | ND | 362 (336, 388) |
| China |  |  |  |  |  |  |  |
| Men (n=733) | 113 (103, 122) | 91 (80, 102) | 9 (7, 11) | 37 (32, 43) ^a^ | ND | 73 (56, 89) | 323 (301, 346) |
| Women (n=733) | 107 (97, 117) | 90 (80, 101) | 9 (7, 11) | 47 (41, 53) | ND | 65 (52, 78) | 318 (296, 340) |
| Indonesia |  |  |  |  |  |  |  |
| Men (n=444) | 45 (35, 56) | 51 (45, 56) | 8 (5, 10) | 82 (63, 101) | ND | ND | 186 (164, 209) |
| Women (n=922) | 49 (41, 57) | 52 (48, 56) | 8 (6, 10) | 71 (60, 82) | ND | ND | 180 (165, 195) |
| Japan |  |  |  |  |  |  |  |
| Men (n=698) | 80 (72, 88) | 148 (141, 155) | 25 (22, 28) | 37 (32, 42) | ND | 165 (146, 183) | 455 (433, 478) |
| Women (n=683) | 88 (78, 99) | 153 (146, 160) | 27 (24, 31) | 44 (38, 50) | ND | 153 (135, 172) | 465 (443, 486) |
| Total population |  |  |  |  |  |  |  |
| Men (n=7580) | 135 (130, 140) ^a^ | 98 (95, 100) | 65 (62, 68) | 88 (84, 91) ^a^ | 2 (2, 3) | 119 (113, 125) ^a^ | 512 (503, 522) ^a^ |
| Women (n=8696) | 145 (140, 150) | 100 (98, 102) | 64 (61, 66) | 93 (90, 97) | 3 (2, 3) | 66 (62, 70) | 469 (461, 478) |

Data expressed as mean (95% CI). Abbreviation: ND, no data. ^a^ P-value <0.05 for men vs. women, with a Bonferroni correction applied during the post-hoc test
